# Long-term, treatment-free survival in select patients with distant metastatic papillary thyroid cancer

**DOI:** 10.1530/EC-14-0097

**Published:** 2014-11-05

**Authors:** Norra Kwong, Ellen Marqusee, Michael S Gordon, P Reed Larsen, Jeffrey R Garber, Matthew I Kim, Erik K Alexander

**Affiliations:** 1 Division of Endocrinology Hypertension and Diabetes, Thyroid Section, Brigham and Women's Hospital, Harvard Medical School, 221 Longwood Avenue, Boston, Massachusetts, 02115, USA; 2 Endocrine Division, Harvard Vanguard Medical Associates, Boston, Massachusetts, USA

**Keywords:** thyroid cancer, metastasis, survival, outcome

## Abstract

Well-differentiated thyroid carcinoma (WDTC) generally has a favorable prognosis. However, patients with distant metastatic disease experience progression of disease with a higher mortality. A subset of patients not previously described may challenge the conventional dogma regarding the progressive nature of all metastatic WDTC. Through analysis of our database, we identified patients with distant metastatic WDTC and persistent, minimally progressive disease. In all patients, persistent metastatic disease was confirmed via tissue biopsy, abnormal PET scan, and/or biochemical elevations in thyroglobulin or antibody levels. Progression of disease was monitored clinically and with repeat imaging. We describe five patients with WDTC and pulmonary metastases, aged 8–43 years at diagnosis. All patients underwent initial surgery and radioactive iodine (RAI) ablation, with some receiving multiple treatments. Persistent pulmonary metastatic disease was confirmed over decades (mean 22 years, range 8–42 years) with minimal progression despite no further treatment beyond thyroid hormone suppression. Persistent disease was biopsy-proven in all patients at a mean of 9.6 years from last RAI treatment. All patients had elevated thyroglobulin or anti-thyroglobulin antibody levels, while three demonstrated metabolically active disease with positive FDG uptake on PET scan, and one patient with persistent radioactive iodine avid pulmonary metastasis 36 years after her last RAI treatment. This case series demonstrates that some patients with distant metastases, even if metabolically active and radioactive iodine resistant, remain stable for decades without further treatment. Clinical awareness of such patients and continual reassessment of disease risk following initial therapy are crucial as aggressive treatment may not be necessary.

## Introduction

Well-differentiated thyroid cancer is common, easily identified, and often curable. While the incidence of thyroid cancer has nearly tripled in the past three decades, mortality rates have remained stable (1, 2, 3). This underscores the widespread success in identifying, treating, and thereby limiting disease-related harm in most affected individuals (4, 5). Yet paradoxically, such features may have simultaneously hindered our ability to prospectively investigate the natural history of this illness and define the optimal extent of necessary treatment. This is true for both localized and metastatic disease. For nearly seven decades, the recommended treatment for biopsy-proven disease has been surgical thyroidectomy, and radioactive iodine (RAI) (^131^I) ablation, followed by thyroid hormone suppression therapy (6, 7, 8, 9). Though there may be benefits to this approach, especially for patients with advanced disease, increasing evidence confirms that such a standardized approach to care may not be equally effective for all patients (10, 11, 12).

For example, attention has recently been focused upon the necessity for (and the optimal dosing of) ^131^I in the treatment of papillary thyroid carcinoma. Two separate prospective trials confirmed the equivalency of 30 and 100 mCi ^131^I dosing for post-surgical remnant ablation (13, 14). At a follow-up of two years, both studies demonstrated effective remnant ablation in 85–90% of patients, depicting lower side-effect profiles, costs, and complication rates when 30 mCi ^131^I was administered. These findings have led to frequent adoption of 30 mCi ^131^I for the treatment of low-risk patients (15, 16). In addition, a recent study has also shown an equivalent efficacy by low-dose RAI on tumor outcome in patients with intermediate tumor risk (17). More broadly, such prospective and randomized data have also raised questions about the necessity, extent, and duration of treatment for all thyroid cancer patients, even those with advanced or metastatic disease. Importantly, there currently exist no randomized controlled trials comparing ^131^I with placebo. As a consequence of these studies, there has been a paradigm shift over the past two decades to individualize risk assessment and treatment strategies in an effort to avoid potential harm, especially in patients with low-risk disease (10, 11, 12, 18, 19, 20).

However, these concepts are more difficult to apply in patients with advanced disease, given their guarded prognosis. The 10-year survival rate for patients with distant metastatic thyroid cancer ranges from 26 to 60% (21, 22, 23). As a result, more aggressive and repeated therapies are often employed. For instance, most patients with pulmonary metastases are considered for repeated RAI treatment so long as iodine avidity is still confirmed. This approach, while seemingly logical, nonetheless remains untested when compared with a more conservative regimen.

Observations have suggested that a subset of such patients may challenge the conventional dogma regarding the progressive nature of all thyroid malignancies with distant metastases. Some patients, who have widely metastatic and seemingly persistent thyroid cancer, demonstrate minimal progression of their disease over years despite no further treatment beyond TSH suppression. Clinician awareness of this unique group is crucial as conventional treatment protocols may not be fully applicable. In this paper, we present a case series of patients with distant metastatic thyroid cancer whose disease remained quiescent without observable progression of pulmonary metastases for decades. While standard initial therapy was administered, such therapy did not eliminate all malignant tissues as proven by biopsies of metastatic foci and evidence of metabolic activity on PET and radionuclide scans. These data suggest that persistent distant metastatic thyroid cancer in some patients can be effectively managed over time with only TSH-suppressive therapy.

## Methods

We searched in the thyroid nodule database of the Brigham and Women's Hospital (BWH) to identify patients with long-standing yet stable thyroid cancer metastases. To expand our search, we also surveyed thyroidologists at BWH and at affiliated institutions. Criteria used for identification included the following. All patients were initially diagnosed with well-differentiated thyroid cancer (papillary or follicular carcinoma), confirmed histopathologically following thyroidectomy. Distant metastatic disease was first identified by imaging at the time of initial staging or during follow-up surveillance. All patients have pulmonary metastatic disease and some have additional regional nodal disease. Importantly, metastatic foci were confirmed by tissue biopsy in all cases. Of the 6665 patients consecutively enrolled in the BWH thyroid nodule database, 791 patients proved to have papillary thyroid carcinoma (PTC). Of these, ∼25–35 patients have been found to have confirmed or suspected distant metastatic disease. Several post-^131^I nuclear scans have shown possible, though not conclusive, iodine uptake at distant metastatic sites. As we cannot definitively confirm metastatic disease without tissue sampling (which many did not receive), we acknowledge a level of uncertainty to this calculation.

All patients received standard therapy for their illness, including thyroidectomy followed by ^131^I administration. All patients were prepared for ^131^I therapy using thyroid hormone withdrawal. Typically, doses of ∼75–200 mCi ^131^I were administered, and post-therapy whole-body scanning was performed 3–9 days thereafter. Patient follow-up and repeat assessments were performed as per the practice of the treating endocrinologist. In general, patients were monitored by biochemical testing including serum thyroglobulin measurement, ultrasound, and cross-sectional imaging. Monitoring generally occurred biannually or annually.

Ideally, the goal of ^131^I therapy was to destroy thyroid follicular cells, including those that were malignant. When this happens, metastatic foci or adenopathy can remain anatomically abnormal due to fibrosis or scarring. In such cases, while malignant cells are not present, imaging may detect persistent abnormality. Our goal was to exclude such cases, as they represent effectively treated disease. Thus, we included only subjects for whom we could identify persistent and/or metabolically active metastatic disease despite initial treatment. For this study, persistent disease was confirmed histologically with lung biopsy at a time distant from initial therapy. Lesions were also noted on cross-sectional imaging and increasing thyroglobulin concentration (or new anti-thyroglobulin antibodies) was commonly detected after decades. Metabolically active metastatic disease was defined as positive FDG uptake on PET scanning or lung radioactive iodine uptake upon radionuclide scanning consistent with metastatic disease. Permission was obtained from the BWH Institutional Review Board to perform this analysis.

## Results

A case series of five patients is described below and in Table 1.

### Patient no. 1

A 45-year-old woman was referred for evaluation of persistent metastatic papillary thyroid carcinoma. The patient was first diagnosed with papillary carcinoma and diffuse pulmonary metastases in 1975 at age eight. Owing to the risks associated with therapy, observation was initially favored. Three years later (age 10 years), chest X-ray demonstrated progression of pulmonary metastatic disease. Near-total thyroidectomy and left neck dissection were performed. Histology reported it as a papillary carcinoma with extensive extrathyroidal involvement invading into the trachea and with extensive cervical lymph node metastases. Shortly thereafter, 80 mCi of ^131^I was administered, followed by a second dose of 150 mCi 5 months later. Post-therapy scans confirmed diffuse pulmonary and left neck uptake consistent with distant metastatic carcinoma. Suppressive doses of levothyroxine were administered.

Thereafter, from age 11 to 45 (34 years), the patient received no further therapy and remained in good health, but demonstrated persistently elevated thyroglobulin levels (range 25–57 ng/ml) on intermittent follow-up. At the age of 45, the patient re-established medical care. Evaluation revealed a serum thyroglobulin level of 25 ng/ml. Chest computed tomography (CT) demonstrated innumerable bilateral pulmonary nodules ranging from 5 to 10 mm. Whole-body scanning performed with ^123^I revealed faint uptake in the left neck as well as 1.5% uptake in each lung field (3% total lung uptake). PET scan confirmed some lung foci to be FDG avid. Ultrasound of the neck was negative for signs of local recurrence. CT-guided core needle biopsy of the pulmonary nodules confirmed persistent metastatic papillary thyroid carcinoma. The patient has been monitored for 2 years, with follow-up testing demonstrating variable thyroglobulin levels (25–40 ng/ml), though without upward trend. Her most recent thyroglobulin concentration was 33 ng/ml. Repeated chest CT revealed no change in nodularity. Aside from chronic, moderate shortness of breath attributed to vocal cord dysfunction, the patient remains asymptomatic despite her persistent, stable, iodine avid pulmonary metastases that have remained untreated for 36 years.

### Patient no. 2

A 28-year-old female presented with a thyroid mass. Evaluation led to total thyroidectomy and neck dissection. Multifocal papillary thyroid carcinoma, up to 3 cm in size, with capsular invasion was diagnosed. Fifteen cervical lymph nodes were positive for local metastatic disease. The patient received 150 mCi of ^131^I revealing extensive (5%) neck uptake highly suspicious for residual nodal disease. No uptake was noted in the lung fields or bony structures. During follow-up, thyroglobulin was persistently detectable with concentrations of ∼19 ng/ml. A CT scan revealed bilateral pulmonary nodules ranging from 2 to 5 mm in diameter. No further treatment was provided at that time. One year later (age 29), the patient underwent a second right neck dissection due to persistent disease. A second 150 mCi dose of ^131^I was administered, with no post-treatment evidence of lung uptake. Two additional neck surgeries were performed at ages 33 and 35 for persistent malignant lymphadenopathy. Since age 29, no further ^131^I or systemic treatment was administered beyond TSH suppression.

The patient remained stable for 11 years with unchanged pulmonary nodules on repeat cross-sectional imaging. Stimulated thyroglobulin ranged from 1.9 to 2.5 ng/ml over the first 6 years, though was once reported as undetectable. However, she later developed anti-thyroglobulin antibodies, which have since persisted. At the age of 40, PET scanning revealed multiple pulmonary nodules that remained unchanged in comparison to CT scans 13 years prior, though one 0.6 mm left lower nodule was FDG-avid. The patient was referred for a diagnostic video-assisted thoracoscopic surgery (VATS) wedge resection of the left pulmonary nodule because of concerns for a possible new malignancy. Histopathology confirmed persistent, metastatic papillary thyroid carcinoma. Currently, at the age of 41, the patient is asymptomatic and fully functional. Her biopsy-proven pulmonary metastases have remained stable for 12 years.

### Patient no. 3

A 41-year-old female sought medical attention for a thyroid mass. Neck ultrasound revealed a 3.8 cm left thyroid nodule and FNA proved cytologically suspicious. A near-total thyroidectomy and left neck dissection were performed. Pathology revealed a 4 cm papillary thyroid carcinoma with extensive invasion into skeletal muscle and subcutaneous soft tissues. The surgical resection margin involved carcinoma and ten cervical lymph nodes were positive for metastases. She received 142 mCi of ^131^I with post-treatment scans revealing only neck (remnant) uptake. Anti-thyroglobulin antibodies were detected after therapy with increasing concentrations 4 years later (age 45), leading to chest CT evaluation. CT imaging demonstrated innumerable bilateral pulmonary nodules, ranging from 1 to 4 mm in diameter. The patient was treated with a second 151 mCi dose of ^131^I, though post-therapy scans demonstrated no pulmonary or neck uptake. The patient underwent diagnostic VATS exploration and wedge biopsy 1 month later (4 years after her initial therapy), because of concerns for a possible new malignancy. Lung histology was consistent with metastatic papillary thyroid carcinoma. No further treatment beyond TSH suppression was provided. The patient has since been followed for 8 years without further treatment. TSH was maintained suppressed. Despite increasing anti-thyroglobulin antibody concentrations up to a level of 1000 IU/ml, her repeat neck ultrasounds and CT scans of the lungs have shown stable pulmonary metastatic disease for 8 years.

### Patient no. 4

A 29-year-old female was observed to have a thyroid mass in 1968. At that time, she underwent near-total thyroidectomy revealing papillary thyroid carcinoma. Three years after initial diagnosis, she underwent right neck radical dissection for metastatic lymphadenopathy. At the age of 33, a chest X-ray showed bilateral pulmonary nodules. A decision was made to treat her with 75 mCi of ^131^I. Post-therapy radionuclide scanning demonstrated no pulmonary uptake. Given the concern for another malignancy, the patient was referred for diagnostic wedge resection of the two right lung nodules. Histopathology confirmed metastatic papillary thyroid carcinoma. After her radioactive iodine therapy at the age of 33, the patient has received no further treatment beyond thyroid hormone-suppressive therapy. Since age 53, the patient has undergone annual CT scans of the lungs revealing persistent, but stable, pulmonary metastases measuring 2–4 mm in diameter without new nodules or progressive enlargement of the existing nodules. Repeat neck sonography has revealed no evidence of local recurrent disease. Anti-thyroglobulin antibodies developed at the age of 59, and concentrations have remained positive, though variable. Despite biopsy-proven pulmonary metastasis, the patient has been asymptomatic and healthy. At the age of 75, patient had stable pulmonary metastases for 42 years.

### Patient no. 5

In 1966, a 43-year-old female underwent hemi-thyroidectomy revealing papillary thyroid carcinoma. No other therapy was recommended at that time. After 12 years, she developed recurrent disease and underwent complete thyroidectomy. She did not receive further treatment except for thyroid hormone suppression and experienced an unremarkable course for the next 34 years until a mediastinal mass was detected at the age of 77 years. A chest CT confirmed a 2.5 cm mediastinal mass as well as numerous micro- and macro-pulmonary nodules measuring 3–12 mm in diameter. A PET scan showed intense FDG avidity in the sternal mass and mild avidity in a left lung nodule. She underwent resection of the mediastinal mass, confirming PTC with tall cell features. The patient was treated with radioactive iodine therapy (203 mCi dose) but demonstrated no pulmonary uptake. Given concerns for possible pulmonary sarcoidosis and a separate malignancy, diagnostic thoracoscopic wedge resection of the left lower lung was performed 2 months later. This confirmed metastatic papillary carcinoma. No further treatment was provided. For the next 8 years, the patient was monitored with CT scans of the chest demonstrating persistent, but stable, pulmonary metastases. Follow-up testing demonstrated variable thyroglobulin levels (2.2–4.8 ng/ml), though without upward trend. She did have recurrent neck metastases that were resected at ages 81 and 85 years respectively. Pathology confirmed metastatic papillary carcinoma. From age 85 to 87, CT scans showed mild enlargement of pulmonary metastasis but stabilization thereafter. At the age of 89, the patient was unfortunately diagnosed with breast carcinoma and biopsy-proven breast cancer metastasis to the liver and bone. She died within 12 months from rapidly progressing breast cancer. At the time of death, the patient had thyroid cancer for over 47 years, with persistent, stable pulmonary metastases disease for 13 years.

## Discussion

The incidence of thyroid cancer has been increasing (1, 3, 24). Most patients demonstrate low-risk, localized disease, which confers an outstanding long-term survival following treatment (4, 5). However, a minority of patients present with distant metastatic disease, often viewed as life threatening. While the majority of deaths from thyroid carcinoma (2) are indeed those with distant metastatic disease (23, 25, 26), our data demonstrate a remarkable diversity in the natural progression of patients with advanced thyroid cancer. We identified five individuals initially diagnosed with well-differentiated thyroid carcinoma (WDTC), who underwent surgery and ^131^I therapy. However, concerns for separate malignancies led to biopsy of metastatic foci at time-points far distant from the initial diagnosis (mean: 19 years). In each case, histopathological analysis confirmed persistent, generally stable pulmonary metastases long after the completion of initial therapy. Persistent disease was further supported by elevated serum thyroglobulin levels in two patients and the development of new (or rising) anti-thyroglobulin antibodies in the other three patients. Abnormal uptake on PET scanning (*n*=3) and/or persistent iodine uptake on radionuclide scanning (*n*=1) confirmed metabolic activity in these persistent, yet stable, foci of cancer. The lack of disease progression was assessed by a careful review of repeat imaging and the subsequent clinical follow-up, while no further therapy was administered beyond thyroid hormone suppression. Together, these data confirm the remarkable ability of select distant metastatic WDTC to persist yet remain stable for decades (mean 22 years, range 8–42 years) despite evidence of metabolic activity.

Our data do not provide metrics allowing us to determine the prevalence of such patients. We estimate that ∼4% of the patients diagnosed with PTC at BWH have distant metastatic disease. However, our study is notable for the inclusion criteria, which mandated lung biopsy and histological confirmation of persistent metastatic cancer at a later time point. While this protocol was necessary to prove that persistent lung nodules were not simply effectively treated (and destroyed) WDTC tissue, these criteria almost certainly underestimate the number of similar cases that may exist. Although studies have reported high mortality rates in patients with metastatic thyroid cancer, those who survived probably shared a fate similar to our population. In support of this, others have estimated that up to ∼30% of patients with metastatic thyroid cancer remain stable during 10 years of follow-up (27). In addition, many patients with thyroid cancer and lung nodules do not undergo invasive lung biopsy or wedge resection. Most of them are simply monitored by serial clinical examinations and chest imaging. We speculate that at our institution, such cases number at least one per year. Extrapolation to a national level allows one to postulate that hundreds of patients may prove to have persistent, yet non-progressive distant metastatic disease.

Though impossible to identify the predictive characteristics from such a small patient population, it is worth pointing out some striking findings. One notable finding was the paucity of iodine uptake on post-therapy scans in the metastases of four patients (patients nos 2–5). Typically, non-iodine-avid distant metastases are associated with worse prognoses. However, those in our series demonstrated stable disease (28, 29). Separately, FDG positivity on PET scan is also known to be associated with unfavorable prognoses (30). However, all three patients with FDG-avid pulmonary metastasis experienced prolonged stability. We also note that the patients described in this series are all women and were relatively young at the time of diagnosis (mean 29 years). It is well recognized that younger patients respond better to initial therapy and experience a longer life expectancy compared with older patients with a similar disease (6, 31, 32, 33). This case series also lends support to long-term TSH-suppressive therapy in patients with distant metastatic thyroid cancer, as all patients were treated solely by this modality for decades. However, we acknowledge that no prospective, randomized study has yet proven the long-term survival benefit of TSH suppression (34, 35, 36).

Very few published reports exist on this topic. Vassilopoulou-Sellin *et al*. (37) reported distant metastatic papillary carcinoma in a 9-year-old female. She was treated with surgery, external radiation therapy, and ^131^I ablation. Long-term follow-up was unremarkable, though a new provider detected a lung nodule at the age of 40. Biopsy demonstrated thyroid carcinoma ultimately proven to be metabolically active and iodine avid. This is remarkably similar to patient no. 1 described above. However, few other comparable reports (18) exist despite epidemiological data confirming young patients (<45 years) with distant metastatic disease may experience a 63–85% 10-year survival rate after initial therapy (33, 38, 39). We are confident that there are many patients who may experience a similar disease course.

These data have important implications for the clinical care of patients with WDTC. Importantly, distant metastatic disease occurs in 2–7% of cases (22, 40, 41, 42) and should be treated aggressively. It is notable that all patients in our series underwent initial therapy including thyroidectomy and neck dissection, followed by ^131^I ablation. Some subjects (patients nos 2 and 5) have substantial or recurrent neck disease that was aggressively treated and required multiple surgeries. Complete resection of the local disease (including adenopathy) appears to be important (15, 30, 31, 34, 43, 44). When resection is not complete following initial surgery, repeated local dissections may prove beneficial (15, 45). It is likely that such therapy was important to their outcome and should be recommended for similar patients. However, the utility of repeated ^131^I dosing, and possible use of systemic chemotherapy should be questioned, with perhaps more judicious decision-making favored based upon continued individual risk assessment and response to treatment, as advocated by the American Thyroid Association (15). In support of this, repeat RAI dosing in the absence of iodine avidity in metastatic disease has been associated with a low efficacy on tumor outcome (46). Furthermore, some patients in our study may have qualified for enrollment in clinical trials investigating the use of tyrosine kinase inhibitors, especially if disease appeared to have anatomically progressed (patient no. 5). However, a decision for TKI for treating advanced or even advancing DTC must be based on continuous assessment of treatment benefit vs possible toxicity. All patients who have completed initial surgery and ablation should be monitored by serial examinations and imaging. Those who remain asymptomatic and show no signs of anatomic progression may be candidates for expectant monitoring. We recognize that some patients will progress. Therefore, we do not advocate that a singular approach be utilized in all patients, but rather individualized therapy be provided to optimize treatment benefit and minimize morbidity.

In conclusion, although the diagnosis of advanced thyroid cancer usually confers a worse prognosis, this case series demonstrates the remarkable ability of some patients with metabolically active distant pulmonary metastases to persist and remain stable for decades without further treatment apart from TSH suppression. Though our data are not sufficiently robust to ascertain the prevalence or predictive characteristics of this cohort, this study may serve to promote physician awareness of such a unique subset of patients and highlight the treatment dilemmas that often arise while managing such patients. We hope that this case series may serve as a platform upon which further studies can build to better understand this complex disease.

## Figures and Tables

**Table 1:** Description of five patients with advanced, well-differentiated thyroid cancer. Each patient depicts the long-term, yet non-progressive nature of persistent, lung metastases following initial surgical therapy and ^131^I ablation. Levothyroxine-suppressive therapy was provided to all patients, with a goal TSH <0.1 mIU/l.

**Subject no.**	**Age at diagnosis**	**Sites of distant metastases**	**^131^I administration** ^a^	**Age at last treatment**	**Duration of stable distant metastases**	**Metabolic activity**	**Status**
1	8-year-old female	Lungs	80 mCi (age 10)	11 years old	36 years	+^123^I uptake (lungs)	Asymptomatic, age 47 years old, with stable pulmonary nodules. Variable thyroglobulin 25–40 ng/dl
150 mCi (age 11)	(^131^I therapy)	+PET activity (lungs)
2	28-year-old female	Lungs	150 mCi (age 28)	35 years old	12 years	+PET activity (lungs)	Asymptomatic, age 41 years old, with stable pulmonary nodules. +Tg Ab with variable concentration
150 mCi (age 29)	(local LN removal)
3	41-year-old female	Lungs	142 mCi (age 42)	45 years old	8 years	NA	Asymptomatic, age 53 years old, with stable pulmonary nodules. +Tg Ab concentrations increasing
151 mCi (age 45)	(^131^I therapy)
4	29-year-old female	Lungs	75 mCi (age 33)	33 years old	42 years	NA	Asymptomatic, age 75 years old, with stable pulmonary nodules. +Tg Ab with variable concentrations
(^131^I therapy)
5	43-year-old female	Lungs	203 mCi (age 77)	85 years old (local LN removal)	>13 years^b^	+ PET activity (lungs)	Died of metastatic breast cancer at the age of 90 years old. Stable thyroglobulin 2.5–4 ng/dl

a Following thyroidectomy.

b No cross-sectional imaging available before the age of 77 years.
